# Editorial: Coping with an AI-saturated world: psychological dynamics and outcomes of AI-mediated communication

**DOI:** 10.3389/fpsyg.2024.1479981

**Published:** 2024-09-16

**Authors:** Anfan Chen, Richard Evans, Runxi Zeng

**Affiliations:** ^1^School of Communication, Hong Kong Baptist University, Kowloon, Hong Kong SAR, China; ^2^College of Digital Transformation, Faculty of Computer Science, Dalhousie University, Halifax, NS, Canada; ^3^Center for Intelligent Communication and Governance, School of Journalism and Communication, Chongqing University, Chongqing, China

**Keywords:** large language models, chatbots, social media, intelligent communication, Artificial Intelligence Generated Content (AIGC), algorithms, psychology, China

## Introduction

Artificial Intelligence (AI) systems are trained on large amounts of data and adopt technologies such as algorithmic technology, machine learning, and natural language processing to enable computers to learn, solve problems, and integrate seamlessly into our daily lives. From voice-controlled virtual assistants, such as Siri and Alexa, to AI-driven social media algorithms, these technologies influence how we interact with information and each other. Against this backdrop, the rise and general acceptance of AI has ushered in a new era of communication, marked by the widespread integration of AI systems that enhance and mediate human interactions. This Research Topic of Frontiers in Psychology examined the multifaceted nature of AI-Mediated Communication (AI-MC), providing important theoretical and practical insights into user perceptions, coping strategies, and the transformative potential of AI across various communication contexts. It investigated the nuanced ways in which AI influences and reshapes human communication, aiming to explain the complex psychological dynamics and outcomes that arise from our interactions with AI. In the following sections, we detail the focus of our Research Topic and their implications.

## The role of AI in interpersonal communication

One of the central themes explored in this Research Topic was the role of AI in interpersonal communication. AI systems have become adept at mimicking human-like interactions, often blurring the lines between human and machine communication. In the study by Tao et al., the authors investigated how individuals perceive and interact with AI-driven communication tools, specifically focusing on AI paintings. Their research examined the extent to which people attribute human-like qualities to AI, a phenomenon known as anthropomorphism. This tendency significantly impacts users' experiences, influencing trust and emotional connection toward these technologies. In addition, Gu examined the impact of AI-modified background music on social media engagement, focusing on the roles of event relevance, lyric resonance, and the origins of AI-generated singers, along with the mediating effects of audience interpretation and emotional resonance. To wit, this study provided important practical insights into how AI-modified music improves users' cognitive and emotional engagement, fostering a stronger connection between content creators and their audiences.

## Coping and adaptation strategies in AI-MC environments

As AI becomes increasingly embedded in our communication practices, individuals must develop new coping and adaptation strategies to navigate these environments. This Research Topic also explored the various ways people adjust to the presence of AI in their lives, including the strategies they use to manage the complexities and challenges associated with AI-MC. Grassini developed and validated the AI Attitude Scale (AIAS-4), a measure designed to evaluate public perceptions of AI. The authors highlighted the importance of digital literacy and critical thinking skills in coping with AI-MC. As users improve their understanding of the capabilities and limitations of AI, they become better equipped to navigate such systems and mitigate potential negative effects.

Additionally, the integration of AI in smart speakers, which use voice interaction to provide services, poses potential risks to user privacy due to the continuous collection of voice data. Feng explored the factors influencing privacy boundary management among smart speaker users. The author identified that personalization positively influences privacy disclosure and boundary linkage but negatively affects privacy control. Privacy concerns have a negative impact on privacy disclosure and boundary linkage, while positively influencing privacy control. It showed that users with higher privacy concerns are less likely to disclose information and more likely to adopt strict privacy controls. Higher levels of privacy literacy are associated with reduced privacy disclosure and boundary linkage, and increased privacy control. These findings have significant implications for the design and regulation of smart speakers and similar AI-driven devices.

## Chatbots and interpersonal communication

The rise of chatbots and similar tools has transformed the way humans interact with information technology. Lee and Hahn investigated a crucial aspect of human-chatbot interaction: the perception of mind in chatbots. The study found that users who implicitly perceive chatbots as having human-like minds are more likely to perceive the chatbots' messages to be effective, particularly when the chatbots provide emotional support. Users who explicitly attribute human-like minds to chatbots also perceive the chatbots' messages as more effective, regardless of whether the support received is informational or emotional. These findings have significant implications for the design of social support chatbots.

## Psychological dynamics of human-machine interactions

Human-machine interactions are characterized by a complex interplay of psychological factors, including perception, emotion, and cognition. This Research Topic investigated the psychological dynamics of these interactions, examining how individuals perceive and respond to AI systems.

For example, Liu et al. investigated how the labeling of Artificial Intelligence Generated Content (AIGC) affects users' perceptions of automated news using electroencephalography (EEG) to measure brain activity. The study found that AIGC labeling significantly reduces the perceived trustworthiness of both descriptive (fact-based) and evaluative (opinion-based) news. This suggests that transparency cues, like AIGC labeling, nudge users to critically evaluate the quality of the information presented. EEG results indicated higher delta, theta, alpha, and beta Power Spectral Densities (PSDs) when AIGC labeling was present, signifying increased cognitive load and attention. These findings demonstrate the importance of transparency in AI-generated news, while the labeling of AIGC is found to not only help in maintaining journalistic integrity but also enhances users' cognitive engagement, prompting them to process information more critically.

In addition, one of the key findings from this Research Topic is the importance of subjective perceptions in shaping user attitudes toward AI (Tao et al.; Liu et al.; Feng; Lee and Hahn). The research shows that users are more likely to accept and trust AI systems that exhibit a degree of autonomy and intelligence. However, there is also evidence of the “uncanny valley” effect, where highly realistic AI can evoke discomfort and unease. This Research Topic explored these psychological dynamics, providing insights into how designers can create AI systems that are both effective and user-friendly. At the same time, the Research Topic highlighted the potential risks and challenges associated with AI-MC. For example, concerns were expressed about the privacy and security of user data, as well as the potential for AI systems to perpetuate biases and stereotypes. In addition, the Research Topic examined the broader societal implications of AI, including the impact on employment, social inequality, and the digital divide.

## Conclusion

This Research Topic offers a comprehensive exploration of the psychological dynamics and outcomes of AI-mediated communication. The studies presented provide important insights into how AI systems are reshaping human interaction, with significant implications for individuals, organizations, and society at large. As we navigate the complexities of an AI-driven world, developing a nuanced understanding of these systems and their impact on our lives is essential for our daily life.

